# The Dual Functions of a Bracovirus C-Type Lectin in Caterpillar Immune Response Manipulation

**DOI:** 10.3389/fimmu.2022.877027

**Published:** 2022-05-18

**Authors:** Xiaotong Wu, Zhiwei Wu, Xiqian Ye, Lan Pang, Yifeng Sheng, Zehua Wang, Yuenan Zhou, Jiachen Zhu, Rongmin Hu, Sicong Zhou, Jiani Chen, Zhizhi Wang, Min Shi, Jianhua Huang, Xuexin Chen

**Affiliations:** ^1^ Institute of Insect Sciences, College of Agriculture and Biotechnology, Zhejiang University, Hangzhou, China; ^2^ Guangdong Lab for Lingnan Modern Agriculture, Guangzhou, China; ^3^ Ministry of Agriculture Key Lab of Molecular Biology of Crop Pathogens and Insect Pests, Zhejiang University, Hangzhou, China; ^4^ Key Laboratory of Biology of Crop Pathogens and Insects of Zhejiang Province, Zhejiang University, Hangzhou, China; ^5^ State Key Lab of Rice Biology, Zhejiang University, Hangzhou, China

**Keywords:** bracovirus, C-type lectin, immunosuppression, hemocytes proliferation, agglutination, hypoimmunity

## Abstract

Parasitoids are widespread in natural ecosystems and normally equipped with diverse viral factors to defeat host immune responses. On the other hand, parasitoids can enhance the antibacterial abilities and improve the hypoimmunity traits of parasitized hosts that may encounter pathogenic infections. These adaptive strategies guarantee the survival of parasitoid offspring, yet their underlying mechanisms are poorly understood. Here, we focused on *Cotesia vestalis*, an endoparasitoid of the diamondback moth *Plutella xylostella*, and found that *C. vestalis* parasitization decreases the number of host hemocytes, leading to disruption of the encapsulation reaction. We further found that one bracovirus C-type lectin gene, *CvBV_28-1*, is highly expressed in the hemocytes of parasitized hosts and participates in suppressing the proliferation rate of host hemocytes, which in turn reduces their population and represses the process of encapsulation. Moreover, *CvBV_28-1* presents a classical bacterial clearance ability *via* the agglutination response in a Ca^2+^-dependent manner in response to gram-positive bacteria. Our study provides insights into the innovative strategy of a parasitoid-derived viral gene that has dual functions to manipulate host immunity for a successful parasitism.

## Introduction

Parasitism is common in the natural world, and the interactions between parasites and their hosts have received much attention for decades (1). In the evolutionary arms races, the host is under the selection to increase its resistance, whereas the parasite tends to improve its success. Parasitoid wasps are a large group of hymenopteran insects, most of which deposit their eggs into the bodies of their hosts, and the hatched progeny develop by consuming and eventually killing the hosts (2). As a result, the hosts have evolved cellular immune defenses against parasitoids, mainly including the formation of a melanized capsule around the wasp egg (also known as encapsulation), to cause parasitoids death (3–5). For successful parasitization, parasitoid wasps have developed different strategies predominantly based on the use of virulence factors to destroy the immune responses of hosts (6–8).

Polydnaviruses (PDVs) are a special group of large double-stranded DNA viruses that are obligatory symbionts with endoparasitoid wasps in the Braconidae and Ichneumonidae families (7, 9, 10). Based on their wasp family association and morphological structure, PDVs are classified into two different genera, *Bracovirus* (BV) and *Ichnovirus* (IV) (9, 11–13). Based on the available genome information from nine BVs and five IVs, the composition features of the virulence genes in PDVs have been identified, which include *V-ankyrin-motif* genes (*ank*), *Cys-motif* genes, *protein tyrosine phosphatase* genes (*PTP*), *BEN domain-coding* genes, *lectin* genes, *histone* genes, *ribonucleases T2* genes, *epidermal growth factor-like* genes (*EGF*), *glycosylated central domain* genes (*Glc*), and some other hypothetical genes that lack any known domains (10, 14–22). Along with parasitoid oviposition, PDVs enter infected hosts, and their virulence genes have been widely reported to disrupt the host encapsulation reaction, which consists of the accumulation of multiple layers of hemocytes around the wasp egg and the simultaneous deposition of melanin, leading to parasitoid death (3, 5). For example, *PTP-H2* of *Micropilitis demolitor* Bracovirus (MdBV), *TnBV1* and *TnBVANK1* of *Toxoneuron nigriceps* Bracovirus (TnBV), and the *Cys-motif* genes of *Campoletis sanorensis* Ichnovirus (CsIV) can decrease the hemocyte population of parasitized hosts by inducing apoptosis and/or programmed cell death events (23–27). Despite the reduction in hemocyte numbers, the following PDV virulence genes can also change the adhesion and/or spreading characteristics of the host hemocytes to suppress the processes of phagocytosis and encapsulation: *PTP-H2*, *PTP-H3* and *Glc1.8* of MdBV; *CrV1* of *Cotesia rubecula* Bracovirus (CrBV); *Mbcrp* with a cysteine-rich trypsin inhibitor-like domain of *Microplitis bicoloratus* Bracovirus (MbBV); *CpBV-PTPs* and *CpBV15β* of *Cotesita vestalis* Bracovirus (CvBV); and the *Cys-motif* genes and *V-innexin* of CsIV (28–34). Recently, several reports have shown that PDV virulence genes also impair the host humoral immune system. For instance, the *CLP* gene family with a leucine/isoleucine-rich C-terminus in CvBV and two *EGF-like* genes in MdBV inhibit melanization of host hemolymph, and *Ank-H4* and *N5* in MdBV and *P-vank-1* in CsIV disrupt the IMD signaling pathway and reduce the expression of antimicrobial proteins (AMPs) (35–39). It is therefore reasonable that parasitoid wasps have evolved the ability to decrease host immune responses for successful parasitization. However, this raises an important concern regarding how parasitized hosts with hypoimmunity survive when they encounter opportunistic infections, such as those invading deadly pathogens.

Lectins are widespread in most metazoan species and share conserved carbohydrate recognition domains (CRDs), which help recognize and bind to a wide range of carbohydrates located on the outside surface of the cell membrane. As such, lectins play roles in the cross-linking of these recognized cells and agglutination (40, 41). C-type lectins (CTLs) belong to a special group of lectin proteins whose activity depend on the presence of calcium ions (Ca^2+^) (42–44). Interestingly, the functions of CTLs are diverse depending on their original source. For instance, CTLs of viruses can modify glycan structures on the surface of host cells and dramatically alter glycosylation, which benefits microbial invasion (45, 46). However, most CTLs of eukaryotes can detect invaders and recognize microorganisms to enhance microbial clearance (47). In addition to two classic properties of CTLs mentioned above, insect CTLs are able to mediate other innate immune responses, including opsonization, nodule formation, phagocytosis, encapsulation/melanization and prophenoloxidase activation (48, 49). Recently, it has been reported that some insect CTLs originated from PDVs through horizontal gene transfer, and the domestication of these CTLs presents new adaptations and confers the host with protection against baculoviruses (50–52). Given the special biological features of PDVs, CTLs from PDVs in parasitized hosts might be necessary for wasp offspring survival. However, their functions are largely unknown.

*Cotesia vestalis* (Hymenoptera: Braconidae) is a solitary endoparasitoid of the diamondback moth *Plutella xylostella* (Lepidoptera: Plutellidae) and a worldwide pest of cruciferous plants (53–55). Our previous studies presented the complete CvBV genome and showed that some viral genes are involved in the destruction of the host immune responses (39, 56, 57). Here, we report that one *CTL* viral gene of CvBV, *CvBV_28-1*, is highly expressed in the hemocytes of parasitized hosts and serves as a dual functional effector. *CvBV_28-1* suppressed the proliferation of host hemocytes, subsequently reducing the number of hemocytes and the encapsulation reaction in host larvae. On the other hand, *CvBV_28-1* performed classical bacterial clearance *via* agglutination to improve the immunity of the hypoimmune hosts to guarantee wasp progeny development.

## Materials and Methods

### Insects and Cell Lines

*C. vestalis* was reared on *P. xylostella* as the host at 25°C with a relative humidity of 65% under a 14:10 light:dark cycle. *P. xylostella* larvae were provided with cabbage, and all adult *P. xylostella* and *C. vestalis* were fed a 20% honey/water (V/V) solution (56). To obtain parasitized host larvae for the experiments, mid-third instar *P. xylostella* larvae were exposed to one single *C. vestalis* female wasp within a 10 mm (diameter) × 80 mm (height) glass vial.

*L. boulardi* (58) was reared on *D. melanogaster* (W^1118^ strain) as the regular host at 25°C with a relative humidity of 50% under a 16:8 light:dark cycle. The newly emerged *L. boulardi* was provided apple juice agar medium. All *Drosophila* strains used in this study were maintained on standard cornmeal/molasses/agar medium at 25°C in 6-ounce square bottom plastic fly bottles.

To obtain *CvBV_28-1* transgenic flies, *CvBV_28-1* with the hemagglutinin (HA) epitope tag was first cloned into the pUAST-attb vector. The transgenic *Drosophila* line carrying the *UAS-CvBV_28-1* gene was obtained by phiC31 integrase-mediated insertion into the attP2 landing-site locus on the 3^rd^ chromosome.

*Drosophila* Schneider 2 (S2) cells were maintained in 60 mm culture dishes in Schneider’s *Drosophila* Medium (Invitrogen) plus 10% fetal bovine serum (FBS) at 27°C under an ambient atmosphere.

### Transcriptome Sequencing and Analysis

*P. xylostella* hemocytes were sampled in TRIzol reagent at 1 h, 6 h, 12 h, 24 h, 72 h and 120 h post-parasitization with three biological replicates at each time points. RNA extraction, construction of the cDNA library and paired-end RNA-seq (Illumina) were carried out by Annoroad Gene Technology Co., Ltd. The transcriptome sequencing data statistics are listed in **Supplementary Table 1**. The CvBV-related reads were taken from each Illumina library by mapping reads to the CvBV genome (14). The CvBV genome index was built using Bowtie (v2.1.0) (59), and paired-end clean reads were aligned to the CvBV genome using TopHat (v2.1.1) (60). Cuffdiff (v2.2.1) (61) was used to calculate fragments per kilobase of exon per million fragments mapped (FPKMs) for the coding genes in each group, and the FPKM was calculated based on the length of the fragments and the read count mapped to each fragment. The sum of the average FPKMs at these 6 timepoints post-parasitization was used to determine the top 50 CvBV transcriptional levels in hemocytes (**Supplementary Table 2**). Cluster analysis of the CvBV transcription pattern was performed *via* the pheatmap R package (v1.0.12) (62), with FPKMs standardized by natural logarithm. Other heatmap plots were generated with GraphPad Prism (v9.0) with the standardized average FPKM from three biological replications.

### Annotation of the *CTL* Gene Family

The Pfam seed database of CRD (PF00059) (63) and the CTLs in *Bombyx mori*, *Manduca sexta* and *Nasonia vitripennis* retrieved from previous studies (64–66) were used as seeds to annotate the *CTL* gene family in *Cotesia vestalis* bracovirus. Most *CTL* genes in full-sequenced PDVs were primarily annotated with BLASTP (http://www.ncbi.nlm.nih.gov/) based on seed the sequences, and additional CTLs were identified as potential genomic loci by TBLASTN (http://www.ncbi.nlm.nih.gov/) and subsequently predicted using FGENESH (67). Data resources are available in the National Center for Biotechnology Information. All obtained CTL sequences were analyzed by SMART to verify the presence of CRD (68). Signal peptides were predicted with SignalP 5.1 (http://www.cbs.dtu.dk/services/SignalP/). Statistics of the annotated CTL protein sequences are listed in **Supplementary Table 4**.

### Sequence Alignments, Phylogenetic Analysis and Spatial Structure Prediction

Annotated CTL protein sequences in the above species were aligned by MUSCLE in MEGA X with the following parameters: maximum iterations = 100 and clustering method (for iterations 1, 2) = UPGMB (69). Phylogenetic analysis was performed by IQTREE with the automated parameters (70). Phylogenetic trees were viewed using FigTree v1.4.4. Spatial structures of all bracovirus CTLs were predicted with the I-TASSER server (71).

### Gene Cloning

Total RNA was isolated from homogenized parasitized *P. xylostella* using TRIzol and reverse transcribed into cDNA using the PrimeScript™ 1^st^ Strand cDNA Synthesis Kit (Takara). The entire coding region of *CvBV_28-1* was cloned and sequenced. Primer sequences are listed in **Supplementary Table 5**.

### Quantitative Real-Time PCR (qRT–PCR)

Total RNA was extracted and then reverse transcribed into cDNA using the ReverTra Ace qPCR RT kit (Toyobo) according to the manufacturer’s protocol. qRT–PCRs were performed in a CFX Connect real-time system (Bio–Rad) with THUNDERBIRD qPCR Mix (Toyobo). Reactions were carried out for 60 s at 95°C, followed by 40 cycles of 15 s at 95°C and 30 s at 60°C. The *Px-β-Actin* gene (GenBank accession number: NM_001309101) and *Px-β-Tubulin* gene (GenBank accession number: EU127912) were used as internal controls, and the relative concentrations were determined using the 2^−ΔΔCt^ method. All the primers used for qRT–PCR in this study are listed in **Supplementary Table 5**.

### Western Blotting

Total protein from approximately 10 transgenic fly larvae was extracted by Minute™ Total Protein Extraction Kit for Insects (Invent) for western blot according to the manufacturer’s protocol. Samples were diluted in 5× Protein Sodium Dodecyl Sulfate Polyacrylamide Gel Electrophoresis Loading Buffer (Sangon), then boiled for 10 min. Proteins were separated in a denaturing polyacrylamide gel and transferred to a polyvinylidene difluoride membrane.

After blocking and washing, membranes were then incubated with primary antibodies against HA-tag (1:2500, Sangon) or primary antibodies against actin (1:2500, CWBIO) for 2 h at room temperature. Membranes were then incubated with secondary antibody horseradish peroxidase conjugated goat anti-mouse IgG (1:5000, Sangon) in Tris-buffered saline with 0.05% Tween-20 for 2 h at room temperature. After five washes, membranes were then incubated with the enhanced chemiluminescence western blotting substrate for imaging (Promega).

### RNAi

For RNAi, a 25-bp RNA oligo was designed based on the sequence of *CvBV_28-1* and synthesized by Sangon Biotech. The sequence is listed in **Supplementary Table 5**. The miRCURY LNA miRNA mimic (siNC, EXIQON 479903-001) was used as a negative control. A total of 5 pmol of siRNA was injected into each mid-third instar *P. xylostella* larva using an Eppendorf FemtoJet 4i Microinjector with the following parameters: injection pressure = 900 hPa and injection time = 0.15 sec (56). Parasitization was conducted 6 h post-injection, and the RNAi efficiency of *CvBV_28-1* in *P. xylostella* hemocytes was detected 6 h post-parasitization by qRT–PCR. At least three biological replicates were performed.

### Hemocyte Measurements

Hemocyte density of *P. xylostella* was detected with a cell counter (CountStar). Briefly, *P. xylostella* larvae subjected to different treatments were carefully rinsed three times with 1× PBS and dried with filter paper before dissection. Then, 0.5 µL of hemolymph was diluted in 19.5 µL of Typan Blue Solution (Sangon), and the mixture was dropped on an exclusive slide for the cell counter. At least three nonoverlapping images of each slide were captured, and the average concentration was converted into cells per µL. As the volumes of parasitized and nonparasitized *P. xylostella* larvae were the same, the index of hemocyte density was considered as an indicator of hemocyte number in *P. xylostella*.

Hemocyte numbers of transgenic flies were counted with a hematocytometer (Watson). Briefly, transgenic fly larvae at 48 h post-parasitization were carefully rinsed three times with 1× PBS and dried with filter paper before dissection. Then, hemolymph of ten individuals was diluted in 20 µL 1× PBS, and 8 µL of mixture was dropped on the hematocytometer. Circulating hemocytes and lamellocytes were shown with GFP (*Hml>GFP*) and fluorescent red (*MsnCherry*) respectively. The circulating hemocyte numbers and lamellocyte numbers of transgenic fly larvae in four boxes of corners were counted under a Zeiss LSM 800 confocal microscope, and the average number of hemocytes per larva was converted into cells per individual.

### Immunohistochemistry

*Drosophila* larvae 48 h after *L. boulardi* attack were used for imaging. They were carefully rinsed three times with 1× PBS and mounted in a drop of glycerol on a glass slide with their dorsal side facing upward. Mounted larvae were kept at -20°C for 20 min before imaging to ensure that the samples were completely fixed. *L. boulardi* eggs were dissected from the *Drosophila* larvae 24 h post-parasitization in 1× PBS, fixed in 4% paraformaldehyde in PBS for 30 min, and rinsed three times with 1× PBST (PBS containing 0.1% Triton X-100 and 0.05% Tween-20). Samples were mounted in ProLong Gold Antifade Mountant with DAPI (Invitrogen). Fluorescence images were captured with a Zeiss LSM 800 confocal microscope.

For EdU labeling, Click-iT EdU staining was performed on hemocytes from live larvae and S2 cells according to the manufacturer’s instructions (Invitrogen). Host larvae hemocytes were dissected in 1× PBS and allowed to adhere on the slides for 20 min. Then, they were stained with 10 mM 5-ethynyl-2-deoxyuridine (EdU) for 1 h at room temperature and fixed in 4% paraformaldehyde in PBS for 15 min after washing twice with 3% BSA in PBS and once with 1× PBST for 20 min. Fresh cocktail was prepared, and each sample was stained for 30 min, followed by washing twice with 3% BSA in PBS and once with 1× PBST for 20 min. Samples were mounted in ProLong Gold Antifade Mountant with DAPI (Invitrogen). Fluorescence images were captured with a Zeiss LSM 800 confocal microscope.

### Cell Transfection

The ORF of *CvBV_28-1* was subcloned into the pAc-V5/His vector (Invitrogen) with the primers shown in **Supplementary Table 5** to generate the pAc-*CvBV_28-1* plasmid. The Kozak sequence (GCCATGG, the G at positions –3 and +4 of translation initiation) was added to the forward primer, allowing efficient and high-level expression of the recombinant protein in S2 cells. To perform transient transfection, S2 cells were seeded on a cell culture slide in 35 mm culture dishes (to 80% confluence) and transfected with 2.5 μg of pAc-*CvBV_28-1* plasmid using a Lipofectamine 3000 Kit (Invitrogen) with pAc-*GFP* plasmid as a control according to the manufacturer’s instructions.

### Recombinant Protein Expression and Purification

The DNA fragment encoding *CvBV_28-1* was subcloned into the pET32a vector and transformed into the *E. coli* strain BL21 (DE3). Expression of *CvBV_28-1* was induced by isopropyl β-D-1-thiogalactopyranoside (IPTG) at a final concentration of 1 mM. Harvested bacterial cells were washed with 1× PBS and lysed by sonication. The *CvBV_28*-1 protein was expressed in its insoluble form and the sediment was resuspended in 1× PBS with 8 M urea and purified using High-Affinity Ni-NTA Resin (Roche) according to the manufacturer’s instructions. The CvBV_28-1 protein was refolded at 4°C with a stepwise decreasing gradient of urea (6 M, 4 M, 3 M, 2 M, 1 M to 0 M urea) in 1× PBS with 5% glycerol, 1% L-arginine and 2% glycine. The protein was analyzed by 12% SDS–PAGE, detected by staining with Coomassie blue, quantified by the Bradford method, and stored at -20°C for further experiments.

### Agglutination Response Assay

To assess the agglutination activity of CvBV_28-1, *E. coli* and *S. aureus* were collected at OD=0.6 by centrifugation at 3500 rpm for 5 mins, stained by incubation with acridine orange (Sangon, 30 µg/mL) for 20 min at room temperature and washed three times with Tris buffer (20 mM Tris-HCl, pH=8.0). The stained pellets were resuspended in Tris buffer at a concentration of 1× 10^9^ cells/mL for the following test.

For the agglutination response assay, CvBV_28-1 protein was diluted to 10 µg/mL in Tris buffer and mixed with the bacterial suspension in equal volumes. The mixtures were incubated for 1 h at room temperature in the presence or absence of 10 mM CaCl_2_. To test the minimum concentration of bacterial agglutination, CvBV_28-1 was serially diluted in Tris buffer at the concentrations of 10, 0.1, 10^-3^, and 10^-5^ µg/mL. After mixing with an equal volume of bacterial suspension, the mixture was incubated for 1 h at room temperature in the presence of 10 mM CaCl_2_. All samples were observed and photographed under a Zeiss LSM 800 laser confocal microscope.

### Survival Rate Assay

*E. coli* and *S. aureus* were grown overnight at 37°C with shaking at 250 r/min. Cultures were centrifuged at 1000 g. The bacteria were resuspended in sterile PBS to achieve an OD of 0.4, and male flies 7 days post-eclosion were injected with 40 nL of the bacterial resuspension with an Eppendorf FemtoJet 4i Microinjector (Eppendorf) and a microcontroller (Narishige). 0.1 µL bacterial resuspension with an OD of 0.02 in sterile PBS was injected into parasitized *P. xylostella* larvae with *CvBV_28-1* knockdown and control at 1 h post-parasitization. Experiments were performed in triplicate (at least 20 individuals per replicate). Injected flies and *P. xylostella* larvae were then kept at 25°C and recorded after 6 h. Flies and *P. xylostella* larvae were transferred to a fresh container of every day, and death was recorded every 12 h.

### Statistical Analysis

All statistical analyses were performed with GraphPad Prism (v9.0) and Fiji2. Data are expressed as the means ± SD. Log-rank tests were used to determine whether the male fly survival curves were significantly different from one another. For comparison of the hemocyte numbers at 6 and 12 h post-parasitization between the parasitized and nonparasitized host larvae, we performed two-way ANOVA followed by Šidák’s multiple comparisons test with Spearman’s test for heteroscedasticity and the D’Agostino-Pearson omnibus test for normality of the residuals. For comparison of the hemocyte numbers and circulating lamellocyte numbers at 48 h post-parasitization between transgenic flies ectopically expressing *CvBV_28-1* in hemocytes (*Hml>CvBV_28-1*) and control, we performed two-tailed unpaired Student’s *t* test. One-way ANOVAs were conducted for other experiments with Tukey’s multiple comparisons test. The intensity of fluorescence signal and the number of positive cells in the images were calculated with Fiji2. Significant values are indicated as *P < 0.05, **P < 0.01 and ***P < 0.005.

## Results

### Two *CTL* Genes of CvBV Are Highly Expressed in Host Hemocytes

**Figure 1:**
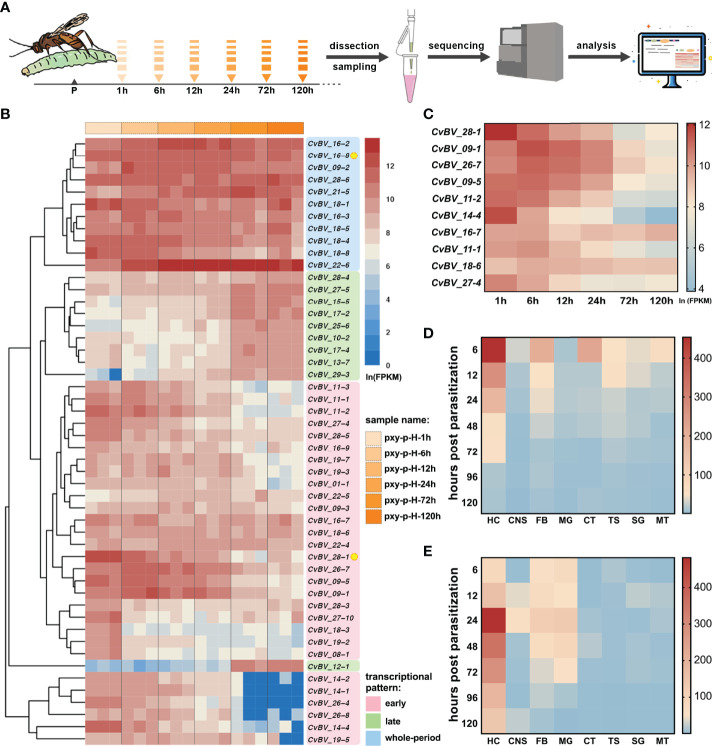
Transcriptional levels of two *CTL*s in parasitized hosts. **(A)** Flow chart of the hemocyte transcriptomes of *C. vestalis*-parasitized *P. xylostella* at different time points, including 1 h, 6 h, 12 h, 24 h, 72 h and 120 h post-parasitization. **(B)** Cluster analysis and transcription heatmap of the top 50 CvBV genes in parasitized host hemocytes. The abscissa (x-axis) represents different transcriptomes at 6 time points post-parasitization with three biological replications, and the ordinate (y-axis) represents the top 50 CvBV genes highly expressed in parasitized *P. xylostella* hemocytes. Values are FPKMs standardized by natural logarithm. Three different transcription patterns are marked with pink, green and blue patches and the relative CvBV gene names. Two *CTL* genes, *CvBV_28-1* and *CvBV_16-8*, are marked with yellow circles. **(C)** Transcription heatmap of the top ten CvBV genes with the early transcriptional pattern in parasitized *P. xylostella* hemocytes. The abscissa (x-axis) represents different times post-parasitization, and the ordinate (y-axis) represents the top ten CvBV genes with the early transcriptional pattern. Values are the average FPKM standardized by natural logarithm with three biological replications. **(D)** Transcription heatmap of *CvBV_28-1* in 8 tissues of *P. xylostella* post-infection. The abscissa (x-axis) represents different tissues including hemocytes (HC), central neural system (CNS), fat body (FB), midgut (MG), cuticular (CT), testis (TS), silk gland (SG) and Malpighian tubule (MT), and the ordinate (y-axis) represents different time points post-parasitization. Values are the average of relative transcriptional level with three biological replications. **(E)** Transcription heatmap of *CvBV_16-8* in 8 tissues of *P. xylostella* post-infection. The abscissa (x-axis) represents different tissues, including HC, CNS, FB, MG, CT, TS, SG and MT, and the ordinate (y-axis) represents different time points post-parasitization. Values are the average of relative transcriptional level with three biological replications.

**Figure 2:**
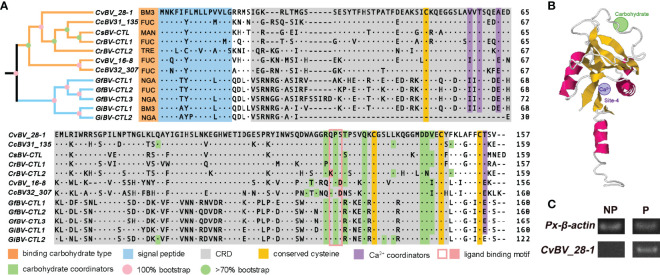
Phylogenetic analysis and spatial architecture of two CTLs of CvBV. **(A)** Multiple sequence alignment and phylogenetic analysis of the CTLs of different PDVs. The gene architecture is shown with a signal peptide in blue, CRD motif in gray, four conserved cysteines in yellow, Ca^2+^ coordinators in purple, a ligand binding motif in pink and carbohydrate coordinators in green. The types of binding carbohydrates are marked in orange. Yellow and blue branches indicate *Cotesia* and *Glyptapanteles* species, with the level of bootstrap in pink and green circles. The numbers on the right are the positions of the final amino acids. BM3, N-acetyl-α-D-mannosamine; FUC, α-L-fucopyranose; MAN, β-D-mannose; TRE, trehalose; NGA, N-acetyl-D-galactosamine. **(B)** Predicted spatial architecture of *CvBV_28-1*. The position of the Ca^2+^ binding site of *CvBV_28-1* is marked in purple, and the carbohydrate binding site is marked in green. **(C)** PCR analysis of *CvBV_28-1* in DNA of parasitized and nonparasitized host larvae. *Px-β-actin* expression was served as control. Representative images from three independent replicates are displayed.

To comprehensively understand the role of CvBV genes in resistance to host cellular immunity, cDNAs were generated from the hemocytes of *C. vestalis-*parasitized *P. xylostella* larvae at a series of time points, including 1 h, 6 h, 12 h, 24 h, 72 h and 120 h post-parasitization. The cDNAs were sequenced using the Illumina HiSeq 2500 platform (**Figure 1A**). The raw sequencing dataset was submitted to the SRA of the NCBI, with the accession numbers from SAMN25185395 to SAMN25185412. After discarding low-quality reads, we obtained clean reads ranging from 39,345,132 to 47,706,030 in these 18 cDNA libraries. The quality Q30 values after data filtering were all greater than 94.19% (**Supplementary Table 1**). Then, we calculated the fragment per kilobase of exon per million (FPKM) values of the CvBV genes in hemocytes using the CvBV genome as a reference. We ranked the top 50 CvBV genes highly expressed in parasitized hemocytes of hosts, and performed the cluster analysis. The transcriptional patterns of CvBV genes in hemocytes were divided into three distinct types: early (higher expression at early time points), late (higher expression at late time points), and whole-period (high expression at all time points) (**Figure 1B**). The details of the gene names and their transcriptional levels are shown in **Supplementary Table 2**. We next focused on the CvBV genes showing high transcriptional levels at early time points in the parasitized hemocytes, because they were most likely to be associated with the host immune suppression process. Among them, we observed one *CTL* gene, *CvBV_28-1*, ranked as the top highly expressed CvBV gene in the early category (**Figure 1C**). The ORF of *CvBV_28-1* is 474 bp, encoding a 157 amino acid (aa) protein with a calculated molecular weight of 17.54 kDa and pI of 7.63 (**Figure 2A** and **Supplementary Table 3**, GenBank accession number: QZB49176.1). *CvBV_28-1* has a predicted N-terminal signal peptide and one single CRD but lacks a typical transmembrane domain, suggesting that it was likely to be a secreted protein into host hemolymph. From the annotated results of the CvBV genome, two *CTL* genes were identified, the other being *CvBV_16-8*, which was placed in the whole-period category. The ORF of *CvBV_16-8* is 471 bp, encoding a 156 amino acid (aa) protein with a calculated molecular weight of 17.55 kDa and pI of 6.45 (**Figure 2A** and **Supplementary Table 3**, GenBank accession number: QZB49081.1). *CvBV_16-8* shares the same sequence feature with *CvBV_28-1.* To further profile a detailed dynamic pattern of the two *CTL*s, we sampled 8 different tissues (hemocytes, central neural system, midgut, fat body, cuticular, Malpighian tubule, silk gland and testis) of *P. xylostella* at different time points post-parasitization and determined the transcription levels of the two *CTL*s by quantitative real-time PCR (qRT–PCR). The results showed that both were highly expressed in hemocytes, with transcriptional level of *CvBV_28-1* being higher at a much earlier parasitism stage and that of *CvBV_16-8* being higher at 24 h post-parasitization (**Figures 1D, E**).

To analyze the sequence features of the CTLs in PDVs, we noted that these two CTLs had a single CRD and a signal peptide at the N-terminus (**Figure 2A**), which is similar to the reported CTLs from other PDVs (72, 73). We further found that *CTL* genes existed only in the bracovirus species of the genera *Cotesia* and *Glyptapanteles* but not in other bracovirus species or ichnovirus species. All of the CTL proteins shared the same domain architecture (**Figure 2A**), including one signal peptide and one single CRD. Interestingly, we found that the number of *CTL* genes was different in the bracovirus species with available genomes. Briefly, one *CTL* gene was identified in *Cotesia sesamiae* BV (CsBV), two *CTL* genes were present in *Cotesia congregata* BV (CcBV), *Cotesia ruficrus* BV (CrBV), *Glyptapanteles indiensis* BV (GiBV) and CvBV, and three *CTL*s were found in *Glyptapanteles flavicoxis* BV (GfBV) with a few different amino acids (**Figure 2A**). Multiple sequence alignment of the BV-derived CTLs separated them into two clusters with high bootstraps, and two pairs of disulfide bonds were found to stabilize the protein structures with four conserved cysteines. The Glu-Pro-Ser motif was identified as the carbohydrate ligand binding motif in the CRDs in the majority of the bracovirus CTLs, while Glu-Pro-Asp existed in only *CvBV_16-8* and *CcBV32_307* and Lys-Pro-Ser existed in only *CrBV-CTL2* (**Figure 2A**). We next constructed the predicted spatial structures of all of these CTLs and found that except for *GiBV-CTL2*, which lacks the Ca^2+^-binding site, all had one Ca^2+^-binding site at site 4, which is important for salt bridge formation between α2 and the β1/β5 sheet (**Figure 2B**) (42, 74). In addition, different types of binding carbohydrates were also predicted in all of these CTLs. As a result, N-acetyl-α-D-mannosamine was predicted in *CvBV_28-1* and *GiBV-CTL1*; α-L-fucopyranose was predicted in *CvBV_16-8*, two *CTL*s of CcBV, *CrBV-CTL1* and *GfBV-CTL2*; β-D-mannose was predicted in one *CTL* of CsBV; trehalose was predicted in *CrBV-CTL2*; and N-acetyl-D-galactosamine was predicted in *GfBV-CTL1*, *GfBV-CTL3* and *GiBV-CTL2* (**Figure 2A**). The details of these CTLs spatial structures are shown in **Supplementary Table 3**. It has been reported that CTLs are widespread in lepidopteran insects and some CTLs from bracoviruses horizontally transferred into genomes of nonparasitized hosts (50, 52), we performed the PCR experiment with specific primers of *CvBV_28-1* using the DNA as template from parasitized and nonparasitized *P. xylostella* larvae. The results showed that *CvBV_28-1* derived from *C. vestalis* bracovirus could be detected in the parasitized hosts, but this gene did not exist in nonparasitized host larvae (**Figure 2C**). Taken together, these results indicate that *CvBV_28-1* may bind to mannose-group carbohydrates on the surface of microorganisms with the help of calcium.

### *CvBV_28-1* Decreases the Hemocyte Numbers in Parasitized Host Larvae and Suppresses the Encapsulation Response

**Figure 3:**
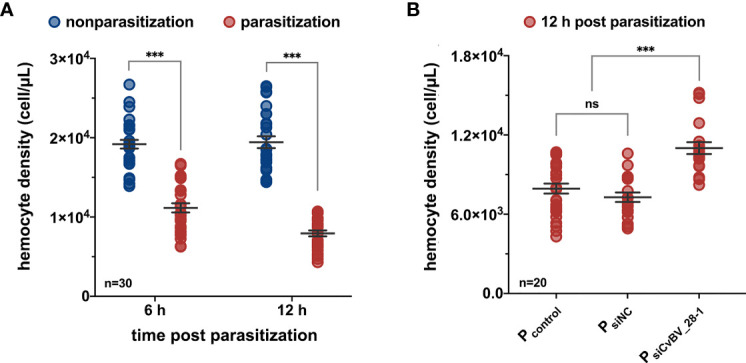
*CvBV_28-1* decreases the number of hemocytes in parasitized *P. xylostella*. **(A)** The hemocyte density of parasitized and nonparasitized *P. xylostella* larvae 6 h and 12 h post-parasitization. 30 independent biological replicates were performed and shown as dots. Data are presented as the mean values ± SD. Differences between groups were analyzed by two-way ANOVA with Šidák’s multiple comparisons test (***p < 0.001). **(B)** The hemocyte density of parasitized *P. xylostella* larvae injected with siCvBV_28-1 (P_siCvBV_28-1_) 12 h post-parasitization. Parasitized *P. xylostella* larvae (P_control_) and siNC-injected parasitized *P. xylostella* larvae (P_siNC_) served as controls. 20 independent biological replicates were performed and shown as dots. Data are presented as the mean values ± SD. Differences between groups were analyzed by one-way ANOVA with Tukey’s multiple comparisons test (***p < 0.001; ns: not significant).

Host defense against parasitoids relies on hemocytes and certain humoral components, which can recognize and respond to invading parasitoids (75–77). However, parasitoid PDVs, contribute to disrupting the resistant responses of the hosts by killing hemocytes or altering their ability to adhere to the surface of invasive foreigners (78). We measured the changes of hemocyte numbers in *P. xylostella* at 6 *h* and 12 h post-*C. vestalis* parasitization. Compared with the nonparasitized larvae, the parasitized hosts showed a significant reduction in the concentration of hemocytes at the early parasitization stage, indicating a decreased number of host hemocytes (**Figure 3A**). To test whether CvBV_28-1 participated in the suppression of host hemocyte numbers because of its early expression profile post parasitization (**Figure 1C**), we next performed RNA interference (RNAi) experiments to knockdown the expression of *CvBV_28-1* in parasitized *P. xylostella* larvae. SiRNAs of *CvBV_28-1* were injected into third instar host larvae, and qRT–PCR showed that the expression of the *CvBV_28-1* gene decreased significantly by approximately 70% (**Figure S1**). We also found that the reduction in hemocytes was effectively suppressed in *CvBV_28-1* knockdown hosts (**Figure 3B**).

**Figure 4:**
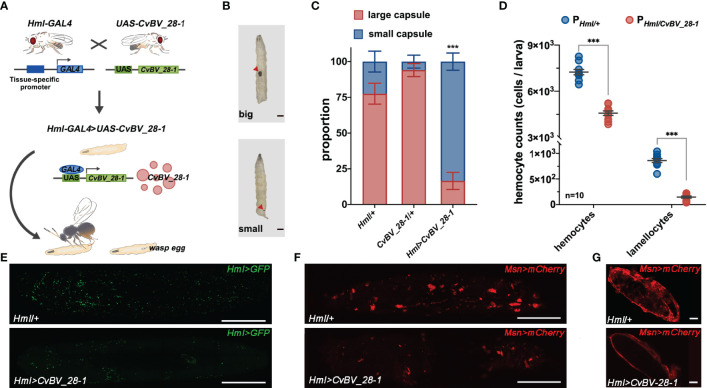
*CvBV_28-1* suppresses encapsulation by reducing host hemocytes. **(A)** A working model of ectopically expressed of *CvBV_28-1* in *Drosophila* hemocytes using the GAL4/UAS system and conducting *L. boulardi* parasitization in transgenic flies. **(B)** Images of the encapsulated phenotypes of *L. boulardi* eggs in *Drosophila* larvae 48 h post-parasitization. Red arrowheads represent melanotic encapsulated wasp eggs. Melanotic encapsulation covered more than 50% of the wasp egg, defined as a large capsule, and less than 50% encapsulation was defined as a small capsule. **(C)** Quantification and phenotypic classification of *Drosophila* larvae ectopically expressing *CvBV_28-1* in hemocytes (*Hml>CvBV_28-1*) 48 h post-parasitization. *Drosphila* larvae with the *Hml-GAL4* driver only (*Hml/*+) and with *UAS-CvBV_28-1* only (+*/UAS-CvBV_28-1*) were served as controls. Experiments were performed in three independent replicates each with 50–55 flies. Data are presented as the mean values ± SD. Differences between groups were analyzed by one-way ANOVA with Tukey’s multiple comparisons test (***p < 0.001). **(D)** The circulating hemocyte and lamellocyte numbers of whole *Drosophila* larva ectopically expressing *CvBV_28-1* in hemocytes (*Hml>CvBV_28-1*) 48 h post-parasitization, circulating hemocytes and lamellocytes are shown with GFP (*Hml>GFP*) and fluorescent red (*MsnCherry*) respectively. *Drosophila* larva with the *Hml-GAL4* driver (*Hml/+*) only served as the control. Ten independent biological replicates were performed and shown as dots. Data are presented as the mean values ± SD. Differences were analyzed by two-tailed unpaired Student’s *t* test (***p < 0.001). **(E)** Image of whole *Drosophila* larva ectopically expressing *CvBV_28-1* in hemocytes (*Hml>CvBV_28-1*) 48 h post-parasitization; hemocytes are shown with GFP (*Hml>GFP*). *Drosophila* larva with the *Hml-GAL4* driver (*Hml/+*) only served as the control. Representative images of three independent replicates are displayed. Scale bars: 500 µm. **(F)** Image of a whole *Drosophila* larva ectopically expressing *CvBV_28-1* in hemocytes (*Hml>CvBV_28-1*) 48 h post-parasitization, and lamellocytes are shown in red (*MsnCherry*). *Drosophila* larva with the *Hml-GAL4* driver (*Hml/+*) only served as the control. Representative images from three independent replicates are displayed. Scale bars: 500 µm. **(G)** Image of a wasp egg dissected from *Drosophila* larva ectopically expressing *CvBV_28-1* in hemocytes (*Hml>CvBV_28-1*) 48 h post-*L. boulardi* parasitization, and lamellocytes are shown in red (*MsnCherry*). A wasp egg dissected from *Drosophila* larva with the *Hml-GAL4* driver (*Hml/+*) only served as the control. Representative images from three independent replicates are displayed. Scale bars: 20 µm.

Consistent with the fact that *CvBV_28-1* can reduce the number of hemocytes, it has been reported that a viral *lectin* gene has the ability to prevent the encapsulation response against wasp eggs (79). Because *P. xylostella* lacks the specific markers for hemocytes and has some difficulties in conducting some certain experiments *in vivo*, we tested the function of *CvBV_28-1 in vivo* using a *Drosophila*-parasitoid system. The parasitoid wasp *Leptopilina boulardi*–host *Drosophila* system is an excellent model and have been widely used to dissect the underlying mechanisms of the parasitoid-induced host immune responses. The encapsulation responses were obvious in *L. boulardi-*parasitized host *Drosophila*, but were absent in the *P. xylostella* and *C. vestalis* system. We then fabricated a GAL4/UAS binary expression system in *D. melanogaster* (80). A transgenic line carrying a UAS transgene encoding the *CvBV_28-1* protein with hemagglutinin (HA) epitopes tagged at the C terminus was constructed. *CvBV_28-1* expression was driven by the specific hemocyte *GAL4* (*Hml-GAL4*), and verified by qRT–PCR and western blotting (**Figures 4A**, **S2A, B**). We then tested the anti-encapsulation response of *CvBV_28-1* in *Drosophila* larvae ectopically expressing viral *CvBV_28-1* from *C. vestalis* in hemocytes. We determined the degrees of the host encapsulation responses and divided them into two categories: large capsules (melanotic encapsulation that covered more than 50% of the wasp egg) and small capsules (melanotic encapsulation that covered less than 50% of the wasp egg) (**Figure 4B**). We found that the proportion of large capsules dramatically decreased, indicating a reduction in host encapsulation responses when *CvBV_28-1* in host hemocytes was ectopically expressed (**Figure 4C**).

We next investigated which cell populations were reduced due to *CvBV_28-1* ectopic expression in *L. boulardi* parasitized hosts. Whole *Drosophila* larvae with different genotypes were imaged, and GFP was used as an indicator of all hemocytes. Overexpressing *CvBV_28-1* reduced hemocyte numbers in parasitized host larvae (**Figures 4D, E**). In addition, *Drosophila* larvae normally generate a special type of hemocyte, called lamellocytes, for encapsulation post wasp parasitization (58, 77, 81, 82). As such, whole larvae with different genotypes were further imaged with MSNF9MO-mCherry (*msnCherry*), a well-known marker for lamellocytes (83). Similar to previous reports, we confirmed that large quantities of lamellocytes were produced 48 h post-*L. boulardi* infection. As expected, overexpressing *CvBV_28-1* led to a significant reduction in circulating lamellocyte numbers in parasitized host larvae (**Figures 4D, F**). We further dissected wasp eggs from the host larvae 24 h after *L. boulardi* attack to determine the encapsulation degree. In comparison with the many lamellocytes adhered to the surface of the wasp eggs, overexpression of *CvBV_28-1* resulted in few lamellocytes on wasp eggs (**Figure 4G**). Collectively, these results indicate that *CvBV_28-1* leads to a significant reduction in circulating hemocytes in parasitized host larvae and prevents lamellocytes from adhering to wasp eggs to initiate the encapsulation response.

### *CvBV_28-1* Inhibits Proliferation of Hemocytes Post-Parasitization

**Figure 5:**
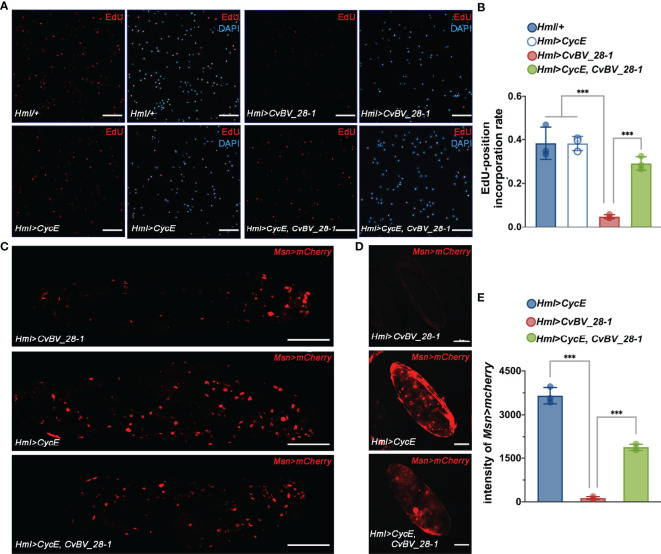
*CvBV_28-1* inhibits hemocyte proliferation in parasitized hosts. **(A)** EdU incorporation assay of hemocytes in *Drosophila* larvae ectopically expressing *CvBV_28-1* and *CycE* in hemocytes (*Hml>CycE, CvBV_28-1*) 48 h post-*L. boulardi* parasitization. *Drosophila* larvae with the *Hml-GAL4* driver only or ectopically expressing only *CycE* or *CvBV_28-1* in hemocytes (*Hml/+*, *Hml>CycE* or *Hml>CvBV_28-1*) served as controls. EdU was stained with EdU-Alexa^594^ (red), and the nuclei were labeled with DAPI (blue). Representative images from three independent replicates are displayed. Scale bars: 50 µm. **(B)** Quantification of EdU staining in hemocytes of *Drosophila* larvae ectopically expressing *CvBV-28-1* and *CycE* in hemocytes (*Hml>CycE, CvBV-28-1*) 48 h post-*L. boulardi* parasitization. *Drosophila* larva with the *Hml-GAL4* driver only or ectopically expressing only *CycE* or *CvBV_28-1* in hemocytes (*Hml/+*, *Hml>CycE* or *Hml>CvBV_28-1*) served as controls. Three independent biological replicates were performed and shown as dots. Data are presented as the mean values ± SD. Differences between groups were analyzed by one-way ANOVA with Tukey’s multiple comparisons test (***p < 0.001). **(C)** Image of a whole *Drosophila* larva ectopically expressing *CvBV_28-1* and *CycE* in hemocytes (*Hml>CycE, CvBV_28-1*) 48 h post-*L. boulardi* parasitization; lamellocytes are shown in red (*MsnCherry*). *Drosophila* larvae ectopically expressing only *CvBV_28-1* or *CycE* in hemocytes (*Hml>CvBV_28-1* or *Hml>CycE*) served as controls. Representative images out of three independent replicates are displayed. Scale bars: 500µm. **(D)** Image of a wasp egg dissected from a *Drosophila* larva ectopically expressing *CvBV_28-1* and *CycE* in hemocytes (*Hml>CycE, CvBV_28-1*) 48 h post-*L. boulardi* parasitization; lamellocytes are shown in red (*MsnCherry*). Wasp eggs dissected from *Drosophila* larvae ectopically expressing only *CvBV_28-1* or *CycE* in hemocytes (*Hml>CvBV_28-1* or *Hml>CycE*) served as controls. Representative images from three independent replicates are displayed. Scale bars: 50 µm. **(E)** Quantification of lamellocytes in *Drosophila* larvae ectopically expressing *CvBV_28-1* and *CycE* in hemocytes (*Hml>CycE, CvBV_28-1*) 48 h post-*L. boulardi* parasitization. *Drosophila* larvae ectopically expressing only *CvBV_28-1* or *CycE* in hemocytes (*Hml>CvBV_28-1* or *Hml>CycE*) served as controls. Three independent biological replicates were performed and shown as dots. Data are presented as the mean values ± SD. Differences between groups were analyzed by one-way ANOVA with Tukey’s multiple comparisons test (***p < 0.001).

To further ascertain the mechanisms of *CvBV_28-1* post wasp parasitization, we proposed that excessive programmed cell death or an impaired proliferation rate may result in a reduction in circulating hemocytes (84, 85). We first examined the level of apoptosis in circulating hemocytes of *CvBV_28-1*-expressing *Drosophila* larvae post wasp infection. Notably, we did not observe a significant difference in the apoptosis level compared with the control groups (**Figure S3**). Additional expression of *DIAP* (Death-associated inhibitor of apoptosis) in hemocytes (*Hml*>*DIAP*, *CvBV_28-1*) also had no effect on inhibiting the reduction in hemocytes (**Figure S4**). We next tested whether the proliferation of hemocytes was impaired *via* 5-ethynyl-20-deoxyuridine (EdU) assays. When *CvBV_28-1* was overexpressed in hemocytes (*Hml*>*CvBV_28-1*), the number of EdU-labeled circulating hemocytes was significantly lower than that in control larvae, indicating that *CvBV_28-1* inhibits the proliferation of host hemocytes post parasitization (**Figures 5A, B**). Moreover, the expression of *CycE* (cyclin E) in hemocytes (*Hml*>*CycE*, *CvBV_28-1*) rescued the proliferation rate (**Figures 5A, B**). Consistent with the rescued proliferation level, the number of lamellocytes in circulation and attached to wasp eggs also increased compared to the control larvae (**Figures 5C-E**).

**Figure 6:**
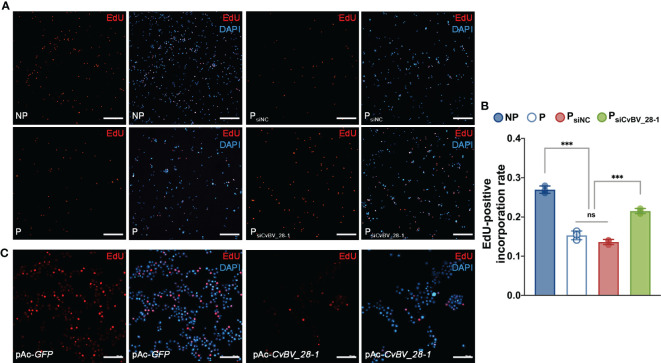
*CvBV_28-1* suppresses proliferation rate of hemocytes in parasitized *P. xylostella* and S2 cells. **(A)** EdU incorporation assay of hemocytes in siCvBV_28-1-injected *P. xylostella* larvae (P_siCvBV_28-1_) 12 h post-parasitization. Nonparasitized *P. xylostella* larvae (NP), parasitized *P. xylostella* larvae (P) and siNC-injected parasitized *P. xylostella* larvae (P_siNC_) served as controls. EdU was stained with EdU-Alexa^594^ (red), and the nuclei were labeled with DAPI (blue). Representative images from three independent replicates are displayed. Scale bars: 50 µm. **(B)** Quantification of EdU staining in hemocytes of siCvBV_28-1-injected *P. xylostella* larvae (P_siCvBV_28-1_) 12 h post-parasitization. Nonparasitized *P. xylostella* larvae (NP), parasitized *P. xylostella* larvae (P) and siNC-injected parasitized *P. xylostella* larvae (P_siNC_) served as controls. Three independent biological replicates were performed and shown as dots. Data are presented as the mean values ± SD. Differences between groups were analyzed by one-way ANOVA with Tukey’s multiple comparisons test (***p < 0.001; ns: not significant). **(C)** EdU incorporation assay of S2 cells transfected with pAc-*CvBV_28-1* and pAc-*GFP*. EdU was stained with EdU-Alexa^594^ (red), and the nuclei were labeled with DAPI (blue). Representative images from three independent replicates are displayed. Scale bars: 50 µm.

Finally, we performed an EdU incorporation assay for circulating hemocytes in *P. xylostella*, the true host of CvBV genes. We found that the ratio of EdU-positive hemocytes clearly decreased, indicating a reduction in the proliferation of circulating hemocytes post parasitization (**Figures 6A, B**). In comparison to siNC-injected parasitized host larvae, silencing *CvBV_28-1* in parasitized *P. xylostella* partially rescued the reduction in proliferation in circulating hemocytes (**Figures 6A, B**). An EdU incorporation assay was also conducted in S2 cells with pAc-*CvBV_28-1* and control (pAc-*GFP*) 48 h post-transfection, and overexpression of *CvBV_28-1* in S2 cells downregulated the EdU-positive rate (**Figure 6C**). These data suggested that *CvBV_28-1* represses hemocyte proliferation to inhibit host cellular immunity after wasp parasitization.

### *CvBV_28-1* Affects *S. aureus* Agglutination and Improves the Survival of Infected Host Larvae

**Figure 7:**
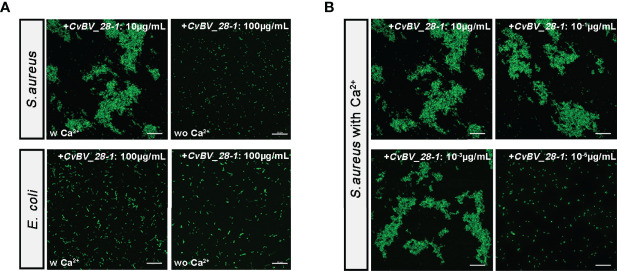
*CvBV_28-1* participates in the agglutination response to *S. aureus*. **(A)** Bacterial agglutination response assay of *CvBV_28-1* in response to *S. aureus* and *E. coli* with or without Ca^2+^. The concentration of *CvBV_28-1* used in each assay is shown in the images. Representative images from three independent replicates are displayed. Scale bars: 20 µm. **(B)** Bacterial agglutination response with different concentrations of *CvBV_28-1* in response to *S. aureus* with Ca^2+^. The concentration of *CvBV_28-1* used in each assay is shown in the images. Representative images from three independent replicates are displayed. Scale bars: 20 µm.

CTLs with CRDs can recognize pathogens by interacting with their cellular surface and promote bacterial agglutination to mediate immune defense responses (42–44). Moreover, our predicted spatial structure of *CvBV_28-1* indicated that it may perform bacterial agglutination with the help of calcium. To test whether *CvBV_28-1* had this ability, we performed an experiment to evaluate the function of *CvBV_28-1* agglutination activity in response to a gram-positive bacterium (*Staphylococcus aureus*) and a gram-negative bacterium (*Escherichia coli*). We found that *CvBV_28-1* showed a strong agglutination response to *S. aureus* in the presence of Ca^2+^ and no agglutination activity to *E. coli* with or without Ca^2+^ (**Figure 7A**). Moreover, the concentration of *CvBV_28-1* resulting in an agglutination response to this gram-positive bacterium was low. In our study, the minimum concentration in a Ca^2+^-dependent manner was 10^-3^ µg/mL (**Figure 7B**).

**Figure 8:**
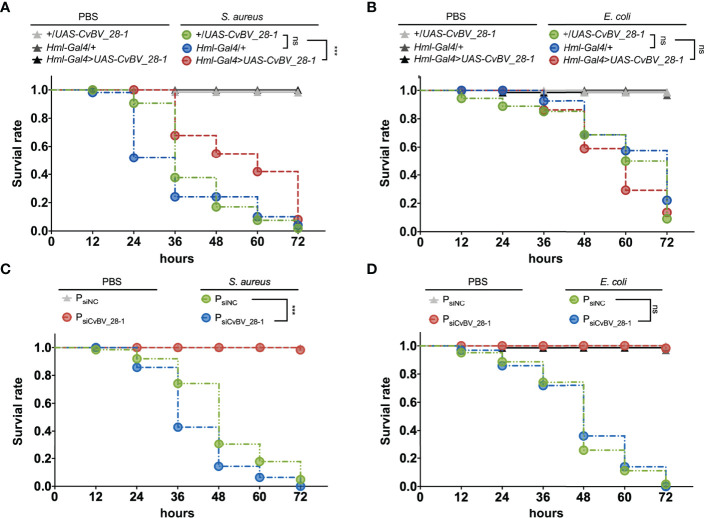
Overexpression of *CvBV_28-1* improves the survival rate after infection with *S. aureus*. **(A)** Survival rates of male *Drosophila* adults ectopically expressing *CvBV_28-1* in hemocytes (*Hml-Gal4>UAS-CvBV_28-1*), with *Hml-GAL4* driver only (*Hml/+*) and with *UAS-CvBV_28-1* only (*+/UAS-CvBV_28-1*) after injection of *S. aureus*. The flies injected with PBS served as controls. Experiments were performed with three independent replicates, and at least 20 flies were used for each replicate. Differences between groups were analyzed by the log-rank test (***p < 0.001; ns: not significant). **(B)** Survival rate of male *Drosophila* adults ectopically expressing *CvBV_28-1* in hemocytes (*Hml-Gal4>UAS-CvBV_28-1*), with the *Hml-GAL4* driver only (*Hml/+*) and with *UAS-CvBV_28-1* only (*+/UAS-CvBV_28-1*) after injection of *E. coli*. The flies injected with PBS served as controls. Experiments were performed with three independent replicates, and at least 20 flies were used for each replicate. Differences between groups were analyzed by the log-rank test (ns: not significant). **(C)** Survival rates of *CvBV_28-1* knockdown in parasitized *P. xylostella* larvae (P_siCvBV_28-1_), with siNC-injected parasitized host larvae (P_siNC_) after injection of *S. aureus*. The parasitized host larvae injected with PBS served as control. Experiments were performed with three independent replicates, and at least 20 *P. xylostella* larvae were used for each replicate. Differences between groups were analyzed by the log-rank test (***p < 0.001; ns: not significant). **(D)** Survival rates of *CvBV_28-1* knockdown in parasitized *P. xylostella* larvae (P_siCvBV_28-1_), with siNC-injected parasitized host larvae (P_siNC_) after injection of *E. coli*. The parasitized host larvae injected with PBS served as control. Experiments were performed with three independent replicates, and at least 20 *P. xylostella* larvae were used for each replicate. Differences between groups were analyzed by the log-rank test (ns: not significant).

To confirm the antibacterial function of *CvBV_28-1 in vivo*, we performed a survival assay to assess the pathogen susceptibility of previous transgenic flies. After injection with PBS, the flies seldom died in the 72 h assay. In contrast, the survival rate of the flies decreased significantly after challenge with both *S. aureus* and *E. coli.* Interestingly, flies overexpressing *CvBV_28-1* in the hemocytes exhibited decreased susceptibility to *S. aureus* leading to a rescued survival rate. Consistent with the *in vitro* results, overexpressing of *CvBV_28-1* did not enhance fly survival after infection with *E. coli* (**Figures 8A, B**). We also conducted the survival assay in parasitized *P. xylostella* larvae challenged by *S. aureus* and *E. coli*, and *CvBV_28-1* knockdown only led to the decreasing survival rate of hosts after *S. aureus* infection, compared with siNC-injected parasitized host larvae (**Figures 8C, D**).

In conclusion, *CvBV_28-1* efficiently performed *S. aureus* agglutination in a Ca^2+^-dependent manner, which led to an antibacterial response. However, no effects were observed on *E. coli* infection.

## Discussion

Parasitoid wasps are widespread on earth, and the evolutionary arms race has promoted them to evolve effective weapons to interfere with host immunity. PDVs are special symbionts of endoparasitoid wasps in the Braconidae and Ichneumonidae families that are involved in disrupting host immune responses to benefit parasitization. In this study, we found that one bracovirus CTL gene (*CvBV_28-1*) presented an extremely high level of expression in host hemocytes at the early stage of parasitization. We further discovered that *CvBV_28-1* suppressed the proliferation of host hemocytes, thereby decreasing the number of host hemocytes and reducing host cellular immunity for successful wasp infection. In addition, the antibacterial ability of *CvBV_28-1* to clear *S. aureus* by agglutination provides another possible strategy to strengthen parasitized host immunity when challenged by gram-positive bacteria.

Insect CTLs participate in immune responses post-infection, including prophenoloxidase activation and cellular phagocytosis (48, 49). Here, we found that *CvBV_28-1* is responsible for immune suppression in response to the encapsulation by reducing the number of host hemocytes. Insect hemocytes from circulating hemolymph and hematopoietic organs are crucial for killing parasitoid eggs (77, 86–88). Plasmatocytes in *Drosophila* and granulocytes in Lepidoptera insects, the main type of hemocytes in healthy larvae, are the first to attach to foreign invaders such as wasp eggs, followed by lamellocytes in *Drosophila* or plasmatocytes in Lepidoptera insect surrounding and encapsulating the coated eggs. Lamellocytes and crystal cells in *Drosophila* are involved in the process of melanization to kill the parasite, plasmatocytes and granulocytes in Lepidoptera insects participated in this biological process (5, 76, 89). In *Drosophila*, lamellocyte hematopoiesis induced by wasp parasitization has been widely studied, and different models for lamellocyte hematopoiesis have been proposed. Progenitors of plasmatocyte lineage and lamellocyte lineage in circulating hemolymph proliferate and differentiate for diverse functional hemocytes generation (58). In addition, the posterior signaling center (PSC) of the lymph gland contains the precursor of hemocytes and produces circulating lamellocytes (88, 90). All plasmatocyte subtypes, lamelloblasts and prolamellocytes are proliferating, while lamellocytes have been considered to be terminally differentiated nonmitotic cells (58, 86, 91). Our study provides the evidence that *CvBV_28-1* reduces the total number of host hemocytes and the encapsulation response post infection. Using an EdU incorporation assay, we found that *CvBV_28-1* is responsible for the decreased proliferation rate of host hemocytes. Moreover, the decreases in proliferation rate and encapsulation responses can be rescued by overexpression of *CycE*, a well-known regulator of the G1/S transition for cell proliferation (92). We also verified the functions of *CvBV_28-1* in both its true host *P. xylostella* (*in vivo*) and in S2 cells (*in vitro*). Some previous studies have suggested that translocation of CTLs into hemocytes might be mediated by the endocytosis-associated or phagocytosis-associated receptors in lepidopteran insects (93–95). Recent studies have also revealed that some proteins belonging to the integrin family have the binding ability to CTLs, and may work as its receptors in invertebrates (96–98). Because integrins are well-known to be responsible for cell adhesion and cell proliferation (99), we speculated that *CvBV_28-1* might translocate into hemocytes and suppress hemocyte proliferation by binding with the integrin family of host *P. xylostella*. Collectively, *CvBV_28-1* served as a powerful virulence factor to regulate host hemocyte proliferation and cellular encapsulation for successful parasitism. Maintenance of circulating hemocytes in lepidopteran larvae are challenged by foreign invaders, which has been attributed to proliferation of circulating hemocytes and the release of hemocytes from the hematopoietic organ (100). In this study, we focused on the function of *CvBV_28-1* in circulating hemocytes of host larvae, which mainly contains plasmatocytes and granulocytes (101). In Lepidoptera, single lineage and dual lineage models have been proposed for the origin of circulating hemocytes. Plasmatocytes are reported to serve as the stem cells that give rise to granulocytes, oenocytoids and spherule cells. While the immunolabeling assays with specific antibodies indicated that granulocytes and plasmatocytes represent two distinct separate lineages (102–104). In addition, it has been reported that all types of circulating hemocytes in lepidopteran insects with the exception of oenocytoids have the proliferated ability (100). Our results showed that knock-down of *CvBV_28-1* in parasitized host larvae could rescue the decreased proliferation rate and number of circulating hemocytes, indicating that *CvBV_28-1* might suppress the proliferation levels of both granulocytes and plasmatocytes in *P. xylostella* host larvae. However, whether *CvBV_28-1* regulates the differentiation rate of hemocytes remains to be determined. In addition, certain PDV genes, such as *PTP-H2* of MdBV and *TnBV1* and *TnBVANK1* of TnBV, have been reported to induce programmed cell death in host hemocytes leading to the suppression of host immunity (23, 25). Our results suggested that *CvBV_28-1* is not the effector for host hemocyte apoptosis. To the best of our knowledge, this is the first time that a PDV gene has been characterized to be associated with the suppression of hemocyte proliferation, but not for cell apoptosis.

CTLs commonly induce cell agglutination, and bacterial agglutination is an important step in clearing infections (40, 42). Several literatures indicate that virulence genes from parasitoid wasps disable vital components of the host immune system, these evidences, along with the suppression of hemocyte proliferation by *CvBV_28-1*, may increase the risk of parasitized hosts with hypoimmunity by opportunistic infections (35, 105–107). Our results also showed that *CvBV_28-1* was efficient for both bacterial clearance and invader defense by producing an agglutination response in a Ca^2+^-dependent manner, especially for defending the typical gram-positive bacterium, *S. aureus*, As such, this *CTL* gene was suggested as a new parasitic strategy for parasitoid survival to enhance the resistance of parasitized hosts when encountering invading pathogens.

Bracovirus CTLs widely exist in the *Cotesia* and *Glyptapanteles* species of the subfamily Microgastrinae. Previous studies have shown that bracovirus CTLs have a closer relationship with Hymenoptera CTLs than other insect CTLs, suggesting that the common ancestor of *Cotesia* and *Glyptapanteles* species in the Microgastrinae subfamily appeared to have a gene transfer from the wasp genome into a bracovirus (39, 50). There are two *CTL*s in the bracoviruses of *Cotesia* and *Glyptapanteles* species. However, only one *CTL* gene was annotated in *Cotesia sesamiae* BV, probably due to imperfect genome information. Moreover, three *CTL*s were found in *Glyptapanteles flavicoxis* BV with highly conserved amino acids; therefore, we assumed that these genes were more similar to the recent duplications. Two CTLs appear in most bracovirus species with high similarity and a primarily conserved ligand binding site that is not typical of the galactose-type QPD motif and mannose-type EPN motif. The specific sequence of the carbohydrate binding site remains the only reliable means for predicting the mannose or galactose group carbohydrate binding ability of CTLs (51). Although it remains difficult to grasp the entire view of bracovirus CTLs through predictions in reference to CTLs from other species in found in databases, we supposed that other bracovirus CTLs may have functions similar to those of *CvBV_28-1.*

In conclusion, we discovered dual functions of a viral CTL, *CvBV_28-1*, in the *P. xylostella* and *C. vestalis* system. On the one hand, *CvBV_28-1* reduces host hemocyte proliferation, which leads to the decreased hemocyte cell numbers and suppresses the host encapsulation response; on the other hand, *CvBV_28-1* possesses Ca^2+^-dependent agglutination activity and antibacterial ability. These findings expand our knowledge and provide insights into the parasitic strategy of PDVs to balance the immune status of the parasitized hosts that benefit parasitization.

## Data Availability Statement

The datasets presented in this study can be found in online repositories. The names of the repository/repositories and accession number(s) can be found in the article/**Supplementary Material**.

## Author Contributions

JH and XC designed and conceived the project. XW performed and analyzed experiments and contributed to all figures and tables. ZWW helped to perform the western blotting and hemocyte measurements. JC helped to perform the plasmid construction. LP and YS helped to perform the Immunohistochemistry and confocal images collection. ZHW and RH assisted to collect transcriptome samples and perform survival rate assay. XY, YZ, and JZ helped to analysis the transcriptome data. JH, XW, and XC wrote the manuscript, and all authors contributed to the article and approved the submitted version.

## Funding

This work was jointly supported by the Key Project of Laboratory of Lingnan Modern Agriculture (NT2021003), Key Program of National Natural Science Foundation of China (31630060), National Key Research and Development Program of China (2019YFD0300104) to XC, the National Science Fund for Excellent Young Scholars (31622048), and the National Science Foundation of China (32172467) to JH, and the National Science Foundation of China (31672079), and Zhejiang Provincial Natural Science Foundation (LR18C140001) to MS.

## Conflict of Interest

The authors declare that the research was conducted in the absence of any commercial or financial relationships that could be construed as a potential conflict of interest.

## Publisher’s Note

All claims expressed in this article are solely those of the authors and do not necessarily represent those of their affiliated organizations, or those of the publisher, the editors and the reviewers. Any product that may be evaluated in this article, or claim that may be made by its manufacturer, is not guaranteed or endorsed by the publisher.
