# Supplementary material for: The Dual Functions of a Bracovirus C-Type Lectin in Caterpillar Immune Response Manipulation
Source: Front Immunol. 2022 May 18;13:877027. doi: 10.3389/fimmu.2022.877027 (PMC9157488; doi:10.3389/fimmu.2022.877027)
Supplement: Supplementary file 1 [file DataSheet_1.docx]

Supplementary Material


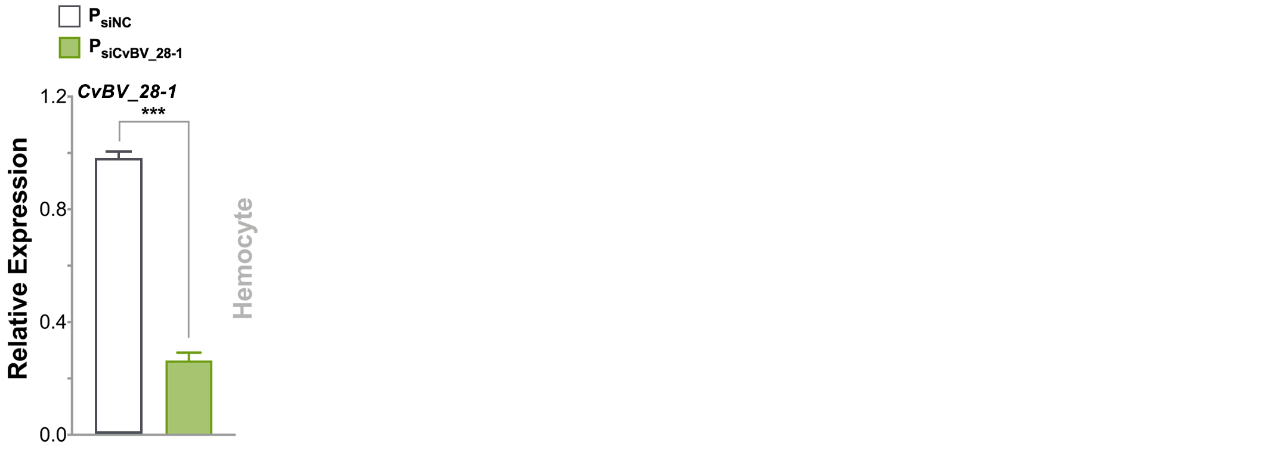


**Supplementary Figure 1.** RNAi Efficiency of *CvBV_28-1* in parasitized *P. xylostella* larvae. Relative mRNA levels of *CvBV_28-1* in host hemocytes 12 h post siCvBV_28-1 injection, with siNC as control. Data are presented as mean values ± SD. Differences between groups were analyzed by two-tailed unpaired Student’s t test (***: p < 0.001).


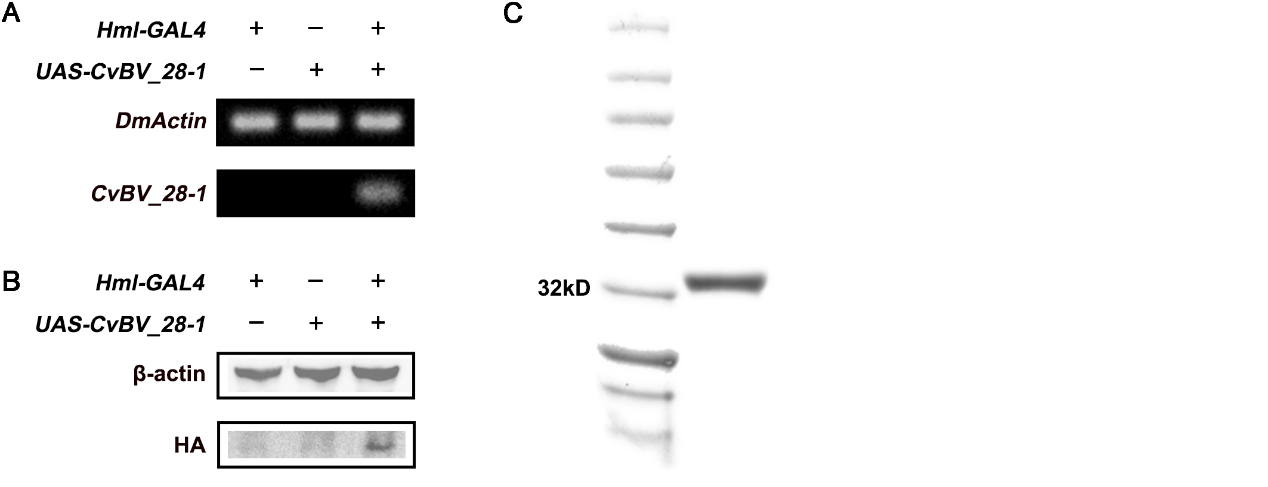


**Supplementary Figure 2.** Confirmation of *UAS-CvBV_28-1* transgenic fly and the purified CvBV_28-1 protein. **(A)** PCR analysis of *CvBV_28-1* expression driven by *Hml-GAL4* in transgenic flies*.* *DmActin* expression was served as control. Representative images from three independent replicates are displayed. **(B)** Western blot analysis of *CvBV_28-1* expression driven by *Hml-GAL4* in transgenic flies. β-actin expression was served as control. Representative images from three independent replicates are displayed. **(C)** Coomassie blue staining of the purified *CvBV_28-1* protein.


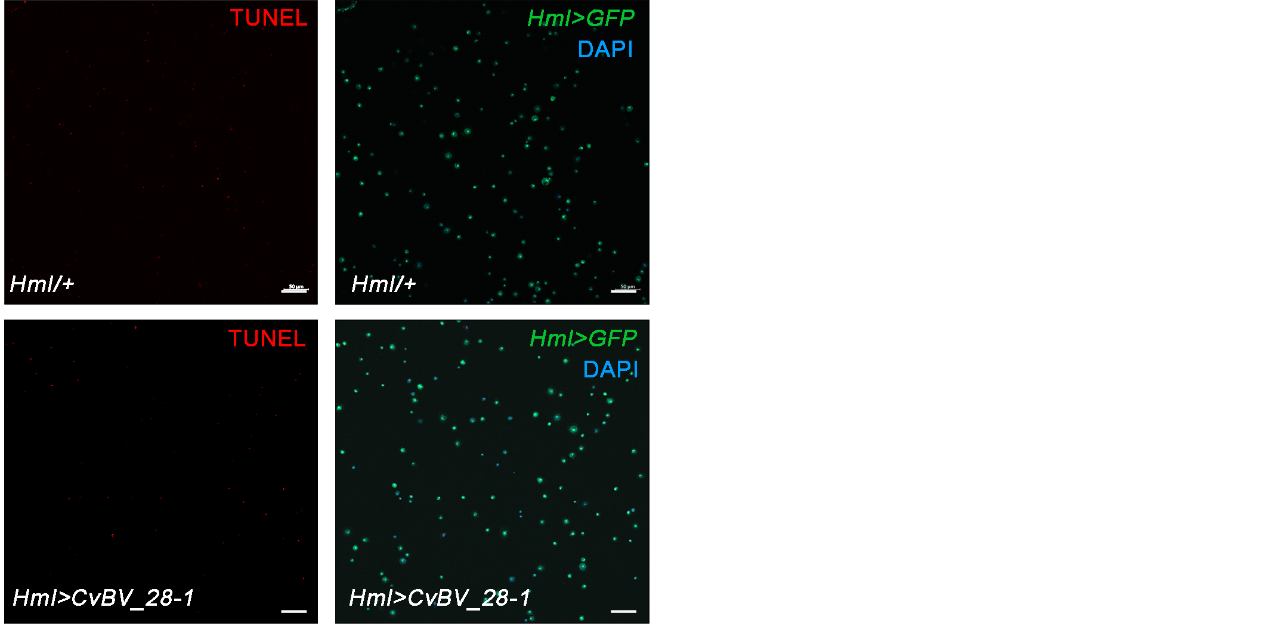


**Supplementary Figure 3.** Apoptosis of hemocytes in *Drosophila* larvae 48 h post-*L. boulardi* parasitization. Cell apoptosis was detected with TUNEL staining (red), hemocytes are indicated with GFP (*Hml>GFP*) and the nuclei are labeled with DAPI (blue). Representative images from three independent replicates are displayed. Scale bars: 50 µm.


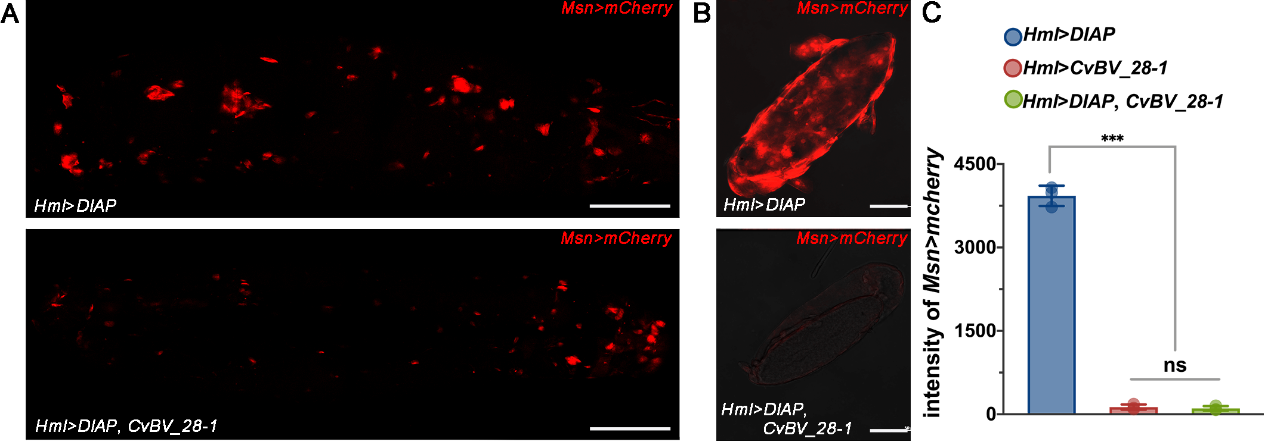


**Supplementary Figure 4.** Overexpression of *DIAP* in hemocytes had no effect on encapsulation reduction. **(A)** Images of whole *Drosophila* larvae ectopically expressing *CvBV_28-1* and *DIAP* in hemocytes (*Hml>DIAP, CvBV_28-1*) 48 h post-*L. boulardi* parasitization, and lamellocytes are shown in red (*MsnCherry*). *Drosophila* larvae ectopically expressing only *DIAP* in hemocytes (*Hml>DIAP*) served as controls. Representative images from three independent replicates are displayed. Scale bars: 500 µm. **(B)** Image of a wasp egg dissected from *Drosophila* larvae ectopically expressing *CvBV_28-1* and *DIAP* in hemocytes (*Hml>DIAP, CvBV_28-1*) 48 h post-*L. boulardi* parasitization; lamellocytes are shown in red (*MsnCherry*). Wasp eggs dissected from *Drosophila* larvae ectopically expressing only *DIAP* in hemocytes (*Hml>DIAP*) served as controls. Representative images from three independent replicates are displayed. Scale bars: 50 µm. **(C)** Quantification of lamellocytes in *Drosophila* larvae ectopically expressing *CvBV_28-1* and *DIAP* in hemocytes (*Hml>DIAP, CvBV_28-1*) 48 h post-*L. boulardi* parasitization. *Drosophila* larvae ectopically expressing only *DIAP* in hemocytes (*Hml>DIAP*) served as controls. Three independent biological replicates were performed and shown as dots. Data are presented as the mean values ± SD. Differences between groups were analyzed by one-way ANOVA with Tukey’s multiple comparisons test (***: p < 0.001; ns: not significant).
